# Design and usability of a system for the study of head orientation

**DOI:** 10.3389/fresc.2022.978882

**Published:** 2022-11-01

**Authors:** Ji Chen, William Geoffrey Wright, Emily Keshner, Kurosh Darvish

**Affiliations:** ^1^ Department of Mechanical Engineering, University of the District of Columbia, Washington, DC, United States; ^2^ Department of Physical Therapy, Temple University, Philadelphia, PA, United States; ^3^ Department of Mechanical Engineering, Temple University, Philadelphia, PA, United States

**Keywords:** multisensory integration, head–neck complex, motion stimuli, acceleration, head mount display, linear track

## Abstract

The ability to control head orientation relative to the body is a multisensory process that mainly depends on proprioceptive, vestibular, and visual sensory systems. A system to study the sensory integration of head orientation was developed and tested. A test seat with a five-point harness was assembled to provide passive postural support. A lightweight head-mounted display was designed for mounting multiaxis accelerometers and a mini-CCD camera to provide the visual input to virtual reality goggles with a 39° horizontal field of view. A digitally generated sinusoidal signal was delivered to a motor-driven computer-controlled sled on a 6-m linear railing system. A data acquisition system was designed to collect acceleration data. A pilot study was conducted to test the system. Four young, healthy subjects were seated with their trunks fixed to the seat. The subjects received a sinusoidal anterior–posterior translation with peak accelerations of 0.06*g* at 0.1 Hz and 0.12*g* at 0.2, 0.5, and 1.1 Hz. Four sets of visual conditions were randomly presented along with the translation. These conditions included eyes open, looking forward, backward, and sideways, and also eyes closed. Linear acceleration data were collected from linear accelerometers placed on the head, trunk, and seat and were processed using MATLAB. The head motion was analyzed using fast Fourier transform to derive the gain and phase of head pitch acceleration relative to seat linear acceleration. A randomization test for two independent variables tested the significance of visual and inertial effects on response gain and phase shifts. Results show that the gain was close to one, with no significant difference among visual conditions across frequencies. The phase was shown to be dependent on the head strategy each subject used.

## Introduction

Falls resulting from postural instability are a major health concern. According to the Centers for Disease Control and Prevention, falls are a leading cause of injury-related death, with more than one-third of adults aged 65 and older falling every year in the United States. The treatment for fall-related injuries is very costly, in part because it often includes hospitalization and long-term care after discharge (1). In 2015, the total direct cost of all fall injuries for people 65 and older exceeded $50 billion (2). Falls also affect the younger population, with approximately 8,000 children treated for fall-related injuries in US emergency departments every day (3).

Postural instability leads to falls in older adults (4–6). Postural stability can be affected by spatial orientation (7). Considerable attention has been directed toward understanding how our visual, vestibular, and somatosensory systems are utilized for spatial orientation. Many factors that contribute to the processes of stability and orientation, including the role of visual context, the dynamic or static state of a subject, the postural state of the subject (sit or stand), the effect of passive vs. self-generated movement, and rotational vs. translational movement, have been identified (8).

The head and neck sensorimotor system is a good prototype for studying whole-body postural control. All three sensory systems are integrated into the central nervous system. In addition, the process of head stabilization is about maintaining an equilibrium position of the head-in-space, and it is accomplished by coordination of head and trunk movements (9). Identifying successful head stabilization strategies is the first step in developing adaptive strategies for fall prevention, movement rehabilitation, and whiplash injury (9).

One way to study head stabilization is to analyze head and trunk acceleration during sled-based locomotion. The participant was seated in the sled that was programmed to provide either position ramp stimuli (10, 11) or jerk perturbation (12–14) in anterior and posterior directions. Kinematics data were collected by using high-end sensors such as 3D motion analysis systems and linear accelerometers. However, the position ramp stimuli only allowed a maximum of 10 cm linear translation (10). Also, jerk perturbation plays little or no role in the genesis of whiplash injuries in low-speed vehicle crashes (13).

Different from the sled system based on the short position ramp stimuli or jerk perturbation, we developed a sled system that can provide up to 3-mlinear and oscillatory perturbation. Our data were collected by only one high-speed camera (2,100 frames per second at the full resolution of 512 × 512 pixels) and lightweight accelerometers. In addition, visual inputs were manipulated by using a head mount display. Our system is expected to serve a similar purpose to a research platform to study the kinematic response of the head to motion-dependent stimuli. The sled system that produces passive motion can be especially useful for examining the combined effects of two kinds of treatments (motion and visual stimuli) on pitch acceleration of the head (15, 16). Our system has been used in a preliminary study investigating how head stabilization is affected by spatiotemporal properties of dynamic visual input when combined with passive motion (17). This paper describes major units of the system and a validation study that investigated whether young healthy participants display different head stabilization strategies in a seated position during passive locomotion.

## Materials and methods

The system can be divided into four units: seating unit, external motion stimuli, visual stimuli, and data collection and processing. The seating unit allowed subjects to be seated with their trunk fixed and head free to move. Trunk fixation was achieved by a five-point harness, which allowed us to minimize the trunk movement and study the head stabilization strategies directly used to interact with the stimuli. It was found that the linear motions of the head imitated the linear motions of the sled when the trunk was fixed, and a fixed trunk also induced higher angular acceleration of the head than when the trunk was free to move (10), which allows the measured acceleration to have a higher signal-to-noise ratio.

External motions and visual stimuli produce inertial inputs and motion-dependent visual inputs to the sensory systems of subjects. By aligning motion with visual inputs concordantly or discordantly, matched or mismatched sensory feedback could be created. Acceleration of the head, trunk, and sled and displacement of the head and sled were collected when the subjects were exposed to external motions and visual stimuli.

### Seating system

**Figure 1:**
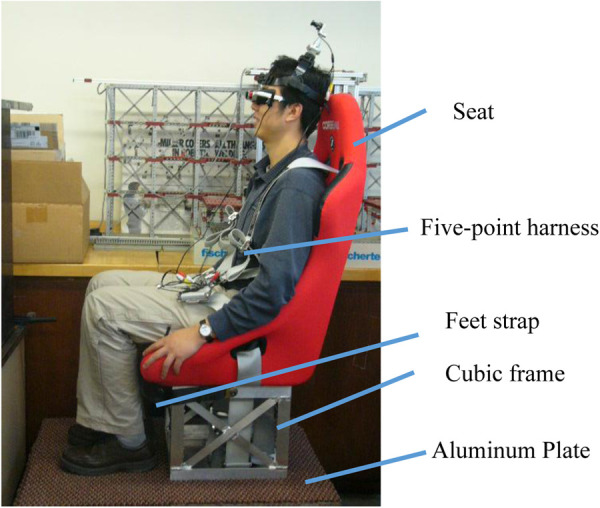
Seating system configuration.

The seating system provided a place to house the subject (Figure 1). We chose a CORBEAU fixed back seat. It has bolster support. It has harness slots for five-point harness capability. A couple of polymer foams was placed between the seat and the subject to create space for the head and neck to move. Five-point harness has a bolt-in feature. Ends of the straps are equipped with a bolt, which allows us to connect them to the linear track. A wrap-around option allowed us to wrap the two rear straps around a harness bar. We also used an extra pluggable strap to restrain the feet during the test. A cubic frame (14.25″ × 13.5″ × 12″) and an aluminum plate (36″ × 24″) provided the connection between the seat and linear track.

### Motion stimuli

A motion system was developed to create the linear translation, which was identified as a sinusoidal motion with adjustable displacements and frequencies. The running cycle of motion is controlled as well. A sinusoidal motion allows for spatiotemporal dynamics to be dissociated between velocity-sensitive visual detectors and acceleration-sensitive inertial detectors and thus can provide insight into how visual and vestibular/proprioceptive sensory systems are integrated into a virtual environment (18).

A sinusoidal shape function was first implemented with a Data Acquisition card (NI-PCI 6281, National Instruments, Austin, TX, United States) to produce the sinusoidal signal and control its parameters such as frequency, amplitude, time length, and triggering for synchronization. It was then output as the single-ended voltage output and filtered through a NI signal conditioning system (SC-2311, National Instruments, Austin, TX, United States). SC2311 has a breaking board that provides two analog-output channels, DAC0 and DAC1. Five-volt DC on-board voltage supply and triggering channels (such as PFI0, PFI1, and PFI2) create the interface for synchronizing the voltage output with the data acquisition system and a high-speed camera. The 5B analog modules provide a signal conditioning solution for data acquisition.

**Figure 2:**
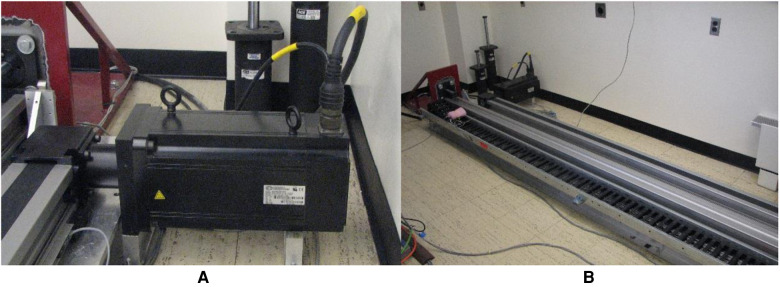
Wh120 linear railing system: (**A**) MH8500 servo motor and (**B**) linear track.

Sinusoidal signals were sent to the Modular Drive System (MDS) (Nidec Motor Corporation, Eden Prairie, MN, United States) with a safety button. An RS-232 Serial Connector provides the I/O communication interface between MDS and PowerTools software (Nidec Motor Corporation, Eden Prairie, MN, United States). PowerTools was used to adjust motor parameters of a linear railing system WH120 (WIESEL SPEEDLine, Thomson, Radford, VA, United States). The motor is geared up to tracks of WH120 whose carriages perform the linear translation (Figure 2). The railing system consists of two 6-m parallel tracks, one is active and transfers the rotation of the motor to the linear motion by a rubber band, and another is passive, with a freely sliding carriage that can move with the active carriage by bolting them together (19).

**Figure 3:**
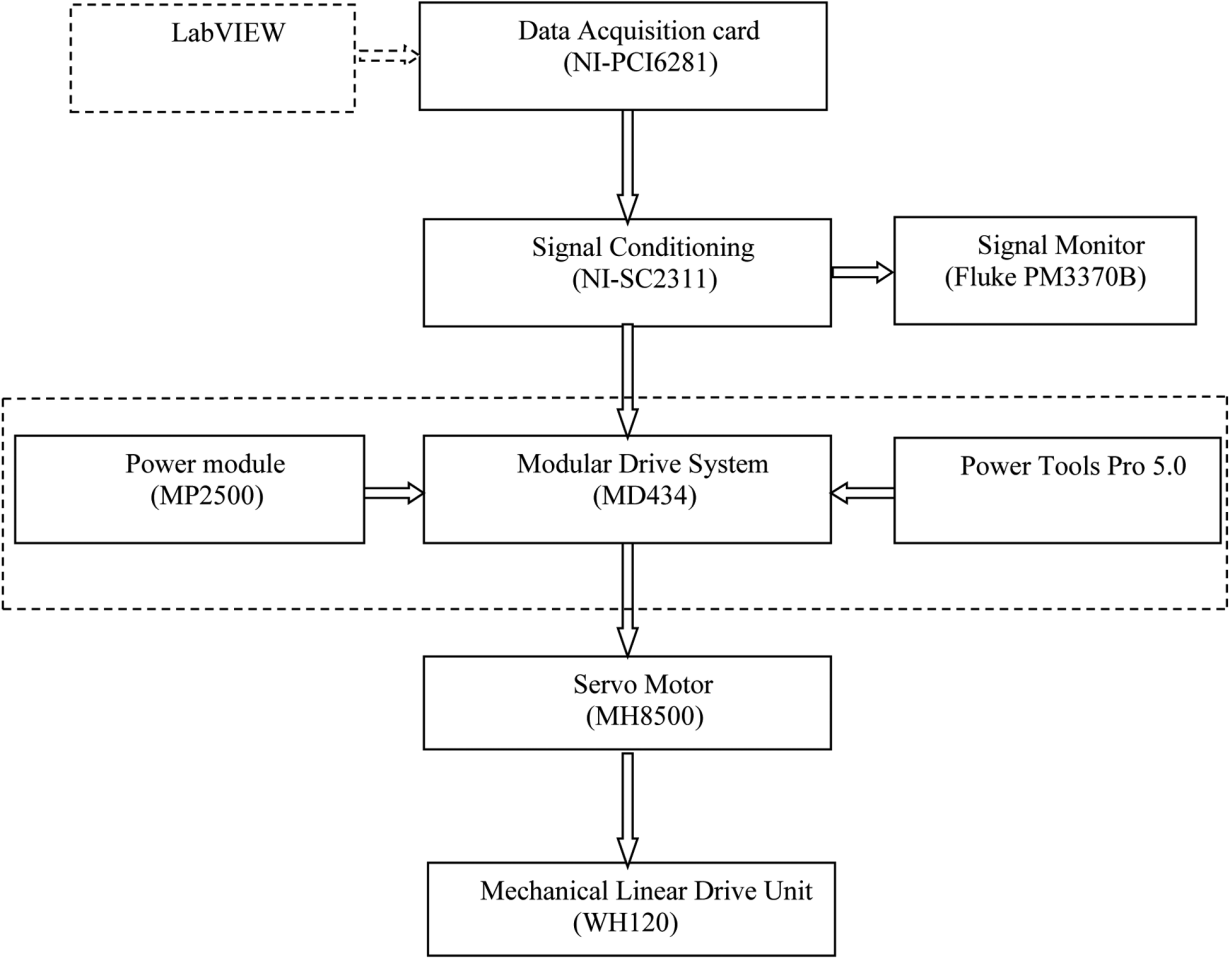
Generation of sinusoidal motion.

The command connector (J5) in MDS was used as the I/O interface to receive external analog signals. Pins 14 and 15 in the J5 connector were physically connected through a cable with a 44-pin D-subconnector to receive the sinusoidal signal. The single-ended sinusoidal signal from these two pins was transformed by MDS to drive the servo motor. The procedure for generating linear translation is described in Figure 3.

**Figure 4:**
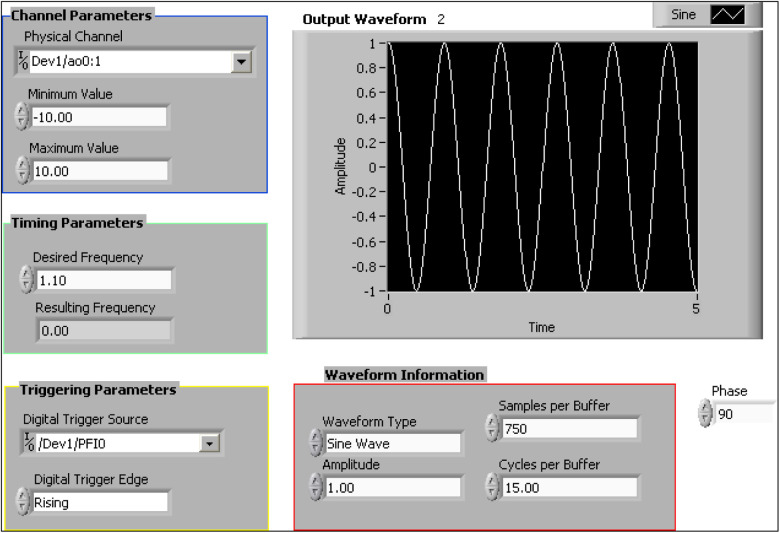
Frontal panel of the LabVIEW program to generate the sinusoidal signal.

The position of the motor corresponds to the voltage input; the maximum voltage represents the maximum position of translation. As the voltage starts from maximum, the motion starts from either end of the track. A 90° phase shift function was added in the sine waveform generator VI in the LabVIEW program to allow the motion sled to start at the end of the track. The frontal panel of the LabVIEW program in Figure 4 shows the sinusoidal signal generation and control parameters. The waveform and frequency of analog input control the motion profile of the carriage through analog position mode in PowerTools. The amplitude of movement is controlled by the amplitude of the signal and its corresponding revolution of the motor in PowerTools. It was also found that the amplitude of movement had an almost linear relation with the amplitude of the sinusoidal signal and the number of revolutions. Due to the limited length of the track, the amplitude of movement with respect to the amplitude of the signal was deduced at the resolution of 7.

### Visual stimuli

**Figure 5:**
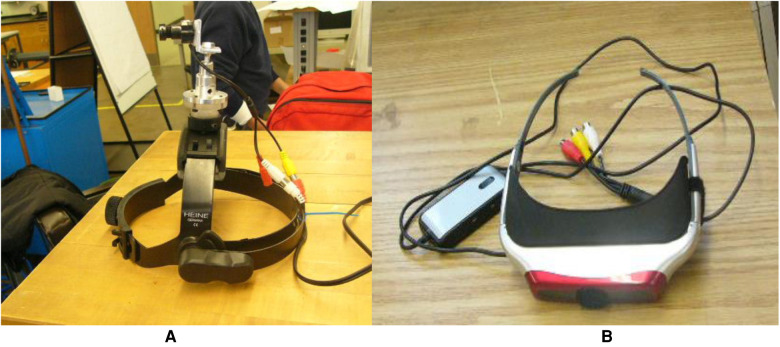
(**A**) Helmet with a gimbaled mini-camera mounted on the top. (**B**) Virtual reality.

A three-dimensional visual field was generated during the experiment to portray a recognizable visual scene. A virtual reality (VR) head-mounted display (HMD) was designed to display the visual scene of the current test environment simultaneously. The HMD consists of a lightweight helmet, a gimbaled mini-camera, and a pair of visual goggles (Figure 5). The camera (203CA-1, Pine Computer, CA, United States) was mounted at the top of the helmet through a holder with multiple degrees of freedom. As a result, the camera was able to provide various directions of live-feed input to the virtual goggles. The helmet also served as the base for attaching accelerometers and photo targets. The VR goggles (I-glasses HR920-3D, 920,000 pixels per LCD, i-O Display Systems, Sacramento, CA, United States) provided the subjects with the required VR inputs taken from the mini-camera. The HMD unit (including VR goggles) weighed less than 400 g. The HMD was light compared to the mass of the head (about 5 kg).

### Acceleration

Head, trunk, and base accelerations were expected to be low, and the head and truck may have both translational and rotational accelerations. Small triaxial accelerometers from Analog Devices (EVAL-ADXL335) were used with an acceleration range of ±3 g and 3 mg resolution at 50 Hz as the motion applied to test subjects was at a very low frequency level (less than 2 Hz). EVAL-ADXL335 is a small, thin, low-cost evaluation platform housing a low-power and complete three-axis ADXL335 accelerometer that measures acceleration with a full-scale range of ±3 g. The EVAL-ADXL335Z weighs 4 g and cost USD 41. We calibrated the accelerometer using the industry standard tumble test from IEEE-STD-1293-1998. The tumble test takes gravity as one reliable source of stimulus for calibrating accelerometers with full-scale ranges of less than 20 g based on the need for the calibration stimulus to equal 5% or more of the full-scale range (20).

The raw output from an accelerometer is a voltage signal. The purpose of the calibration was to derive a linear relationship between voltage and acceleration, as shown in Equation (1),

(1) $$a_{g}=ma_{v}+b$$

where $a_{g}$ is the acceleration in the unit of *g*, $a_{v}$ is the acceleration in the unit of *V, m* is the linearity, and *b* is the offset.

With recorded measurements of x-axis acceleration at four positions relative to the gravity direction in the vertical plane formed by the x-axis and gravity axis (namely, the x-axis is 0°, 90°, 180°, and 270°with regard to the gravity axis), we can find *m* and *b* by Equations (2) and (3),

(2) $$m=\frac{1}{2}[a_{g}(90^{\circ })-a_{g}(270^{\circ })]$$

(3) $$b=\frac{1}{2}[a_{g}(0^{\circ })+a_{g}(180^{\circ })]$$

The values of *m* and *b* would serve as conversion factors for the data acquisition system. As a result, the acceleration value was directly collected in the unit of *g*. At last, we verified the measurement of calibrated accelerometers by the idea that flipping over the direction of the accelerometer over 180°(aligned with the gravitational direction) would cause a 2*g* difference.

### Motion tracking system

The motion tracking system includes a high-speed Phantom camera (2,100 frames per second at the full resolution of 512 × 512 pixels) (Vision Research, Wayne, NJ, United States) and software. The Phantom camera was placed transversely to record the displacement of subject in the longitudinal axis. To determine the lens focal length (FL), a formula is applied to calculate the FL,

(4) $$\mathrm{FL}=\frac{\mathrm{CMOS}\times \mathrm{WD}}{\mathrm{FOV}}$$

Focal length (FL) is the distance between the camera sensor and the center of the lens. The greater the focal length, the larger the image will appear. Field of view (FOV) is the size of the area to be imaged. In our experiment, at least 3 m field of view is required to be covered to record the whole sinusoidal motion at 0.1 Hz. Working distance (WD) is the distance from the camera lens to the area under surveillance. CMOS is the size of the camera's image sensor device.

We have 1″ C-mount CMOS camera. The limited space of the experimental environment does not allow us to change WD on a large scale. Both WD and FL have to be adjusted to achieve the 3 m clear coverage. The displacement of the subject during each test is recorded and saved by Phantom software.

### Data collection and processing

We were interested in the amplitude, phase, and offset of the acceleration of the head, trunk, and seat, as well as the displacement of the head and seat. To remove the noise from the motor and environment coupled into the acceleration measurement, and given that the head–neck system could be considered as a second-order system with linear constant coefficients, a linear regression model was used to smooth the acceleration and to estimate parameters such as amplitude, phase, and offset of the acceleration. To investigate its frequency components, the predicted acceleration underwent the fast Fourier transform.

Data processing also achieved the frame transformation. The acceleration of the sled was measured under the world frame. The acceleration of the head was measured under the local frame. There was an initial rotation angle between the world frame and the local frame. A transformation matrix was used to transform the acceleration of the head from its current frame to the world frame. The acceleration of the head is a vector in space. A transformation law for Cartesian components of vectors was used to transform the vector to the world frame (shown in Equations. (5)–(7)) (21):

(5) $$[a]=[Q][a{]}^{\prime }$$

where $[a{]}^{\prime }$ is the acceleration of the head under the local frame, [a] is the acceleration of the head under the world frame, and [Q] is the transformation matrix.

To find [Q], we have

(6) $$\begin{array}{rl} & {e^{\prime }}_{x}=e_{x}\cos\theta +e_{z}\sin\theta \\ & {e^{\prime }}_{y}=e_{y}\\ & {e^{\prime }}_{z}=-e_{x}\sin\theta +e_{z}\cos\theta \end{array}$$

Then, we get

(7) $$[Q]=\left[\begin{array}{ccc} \cos\vartheta & 0 & -\sin\theta \\ 0 & 1 & 0\\ \sin\theta & 0 & \cos\theta \end{array}\right]$$

The displacement of movement was obtained by using an image processing technique. Motions of the head and seat were recorded as film clips by the Phantom camera. The high-resolution film was converted to images by Phantom software. Vision Assistance software was used to calculate the displacement of markers placed on the head and the tip of seat. The feature of batch processing in Vision Assistance helped obtain the displacement from thousands of images in a very short time. However, due to the limited field of view caused by the space and size of the lens, the camera only captured part of the movement at certain frequencies. All data, such as the amplitude and phase of acceleration and the displacement, were finally put into EXCEL sheets for further investigation.

### System validation

**Figure 6:**
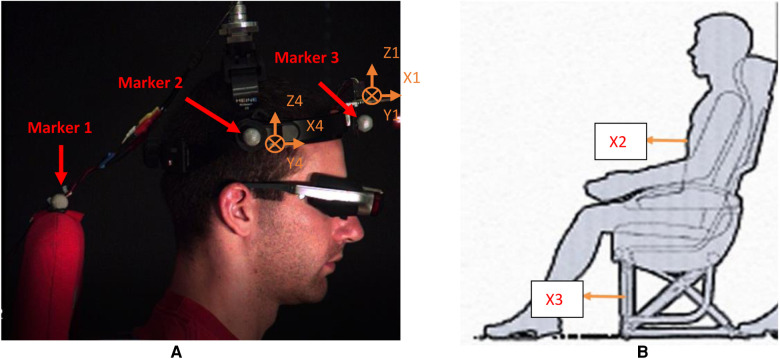
Acceleration data collection. (**A**) Accelerometers 1 and 4 on the head. (**B**) Accelerometer 2 on the torso and accelerometer 3 on the sled. Cross lines at 1 and 4 mean that their Y directions point inward.

The motion of the head–neck complex mainly occurs in the midsagittal plane when the entire body in the sitting posture is exposed to anterioposterior (A-P) translations (15, 22). The focus of the validation study was to examine the reliability of the system that aimed to investigate the combined effect of A-P translation and visual perturbations on head movement. Four male, young, healthy subjects (22–26 years old) were recruited to the validation study and gave signed consent to our approved IRB. Accelerometers No. 1 and No. 4 attached to the head-mounted display (No. 1 is on the frontal site, No. 4 is on the temporal site) measured local head accelerations. Accelerometer No. 2 was attached to the chest to measure trunk acceleration. Accelerometer No. 3 was attached to the cubic frame to measure the acceleration of the sled. Photo markers were placed to record the displacement of the head and seat, as shown in Figure 6. To comply with an anatomical description of directions, the A-P direction represents the X direction, the mediolateral (M-L) direction is the Y direction, and the up-down (U-D) is the Z direction. The angular acceleration of the head–neck system was derived from linear acceleration in related directions.

Motion stimuli in the study were configured with the following four sinusoidal frequencies (0.1, 0.2, 0.5, and 1.1 Hz). The repeatability of the system has been tested to make sure that the programmed sled motion was delivered at the designated frequency and amplitude (as shown in Table 1). The magnitude of acceleration at each frequency was not only tolerated by subjects but also large enough to activate the sensitivity of vestibular/proprioceptive receptors (23).

**Table 1:** Configuration of motion stimuli.

Freq. (Hz)	Angular freq. (w)	Amp. of sine wave (V)	Revs. of motor	Amp. (m)	Accel._max_ (g)	Accel._max_ (m/s^2^)	Vel._max_ (m/s)
0.10	0.63	8.2	7	1.500	0.060	0.59	0.942
0.20	1.26	4.1	7	0.750	0.120	1.18	0.942
0.50	3.14	5	1	0.120	0.120	1.18	0.377
1.10	6.91	0.9	1	0.024	0.117	1.15	0.166

Due to the limited length of the sled and safety concerns, peak acceleration was equalized between 0.2, 0.5, and 1.1 Hz, along with equalized peak velocities at 0.1 and 0.2 Hz. By controlling the acceleration parameters of the input, we have kept the sensitivity of vestibular/proprioceptive receptors the same so that the effect of visual receptors to head motion can be studied.

The frequency of passive motion was configured to be near the cutoff frequencies of visual, vestibular, and somatosensory systems to test sensory dominance associated with postural instability. It was found that the center of pressure signal was distinguished by visual (<0.1 Hz), vestibular (0.1–0.5 Hz), and somatosensory (0.5–1.0 Hz) systems according to the frequency (24).

There were two independent variables for the study, frequency of the sine wave and direction of visual inputs, and one dependent variable, pitch acceleration. Motion stimuli can be configured into four sets: (1) EO—eyes were open and VR was in phase to A-P translation; (2) SW—VR was aligned with the M-L direction; (3) BW—VR was 180° out of phase to A-P translation; and (4) EC—eyes were closed and VR was turned off.

Each level from two treatments was combined, and 16 experimental conditions were created. Each condition was repeated twice to validate the measurement. A total of 48 runs were performed. The orientation of the head was measured before every trial to ensure the head remained in its initial orientation. Angles within ±5% variance met the criteria that the initial orientation of the head did not change.

## Results

**Figure 7:**
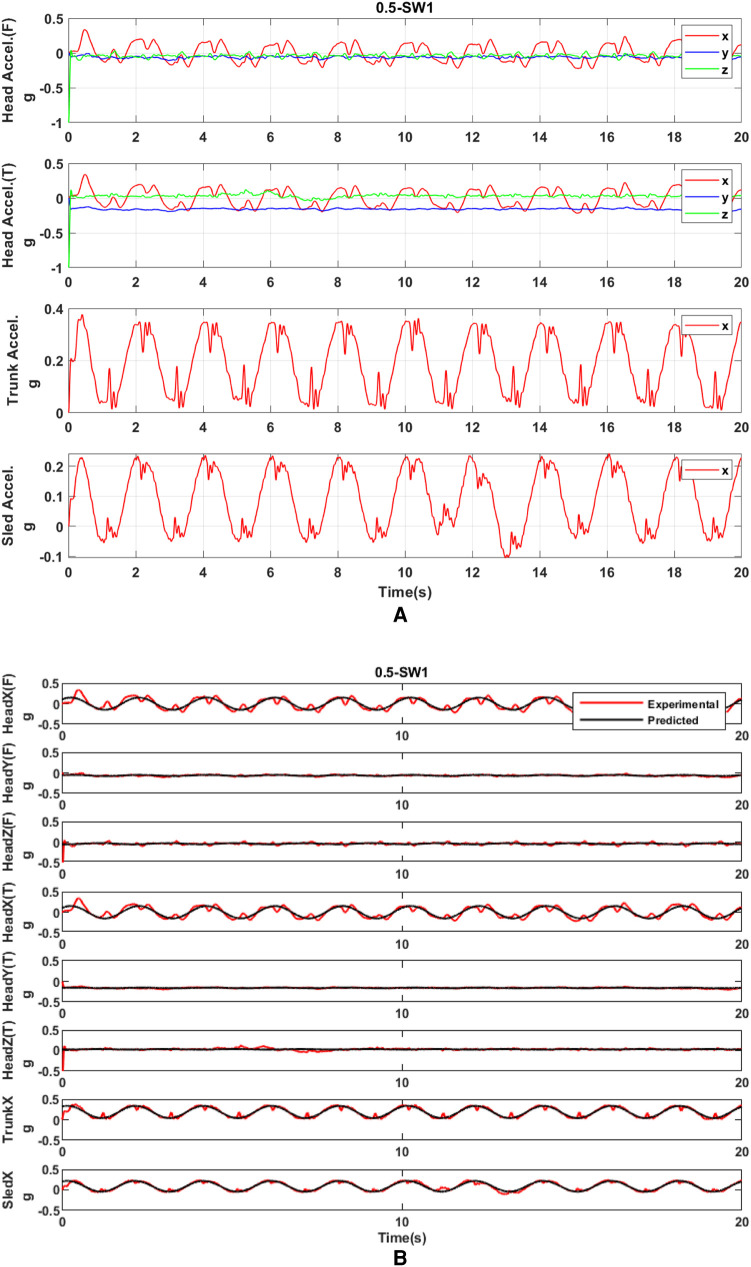
(**A**) Raw acceleration of the head, trunk, and sled with SW at 0.5 Hz. (**B**) Acceleration processed by the fitted-sine model with SW at 0.5 Hz. T and F on Y-axis labels mean the acceleration measured at the frontal part of the head and the acceleration measured at the temporal part of the head, respectively.

**Figure 8:**
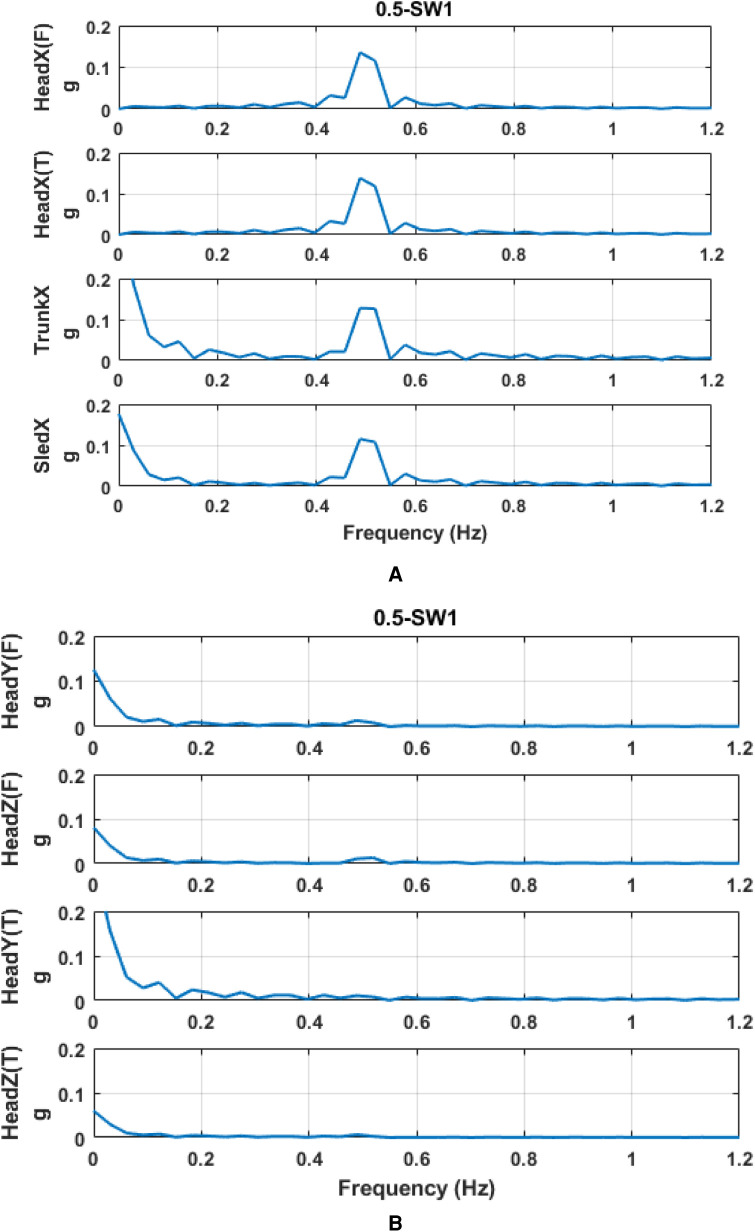
Frequency spectrum of acceleration at 0.5 Hz and SW. (**A**) principal direction and (**B**) secondary direction. T and F on Y-axis labels mean the acceleration measured at the frontal part of the head and the acceleration measured at the temporal part of the head, respectively.

Raw acceleration was processed by the linear regression model for parameter prediction. The *r*^2^ coefficient of the linear regression model was used to decide how well the acceleration pattern fitted the sine curve. The fitted-sine curve was considered to represent well the acceleration pattern if *r*^2^ was larger than 0.5. Compared to raw acceleration data, these sine curves represent well the real acceleration (Figure 7). Then, based on the predicted acceleration, we found the amplitude and phase of linear peak acceleration in all measured directions. Frequency analysis (Figure 8) showed that there would be no harmonics in the dynamic response of the head in both principal and secondary directions, along with all motion profiles and visual conditions. Compared to the acceleration in the principal X (A-P) direction, the acceleration in the secondary Y (M-L) and Z (U-D) directions was very small.

Trunk acceleration had a similar magnitude and was in phase with sled acceleration. With respect to sled acceleration, the gain and phase shift of trunk acceleration were calculated at 1.14 ± 0.06° and −0.82 ± 1.57°, respectively.

The pitch acceleration of the head was also calculated (Equation 8). The frequency response of head pitch acceleration was studied and plotted with the acceleration of the sled. Gains and phases of head acceleration related to the sled were investigated as a secondary interest,

(8) $$a_{\mathrm{pitch}}=\mathrm{sign}(a_{A\text{-}P})\cdot \sqrt{a_{A\text{-}P}^{2}+a_{U\text{-}D}^{2}}$$

Note: As a vector, the direction of $a_{\mathrm{pitch}}$ depends on the direction of $a_{A\text{-}P}$, which is decided by $\mathrm{sign}(a_{A\text{-}P})$.

**Figure 9:**
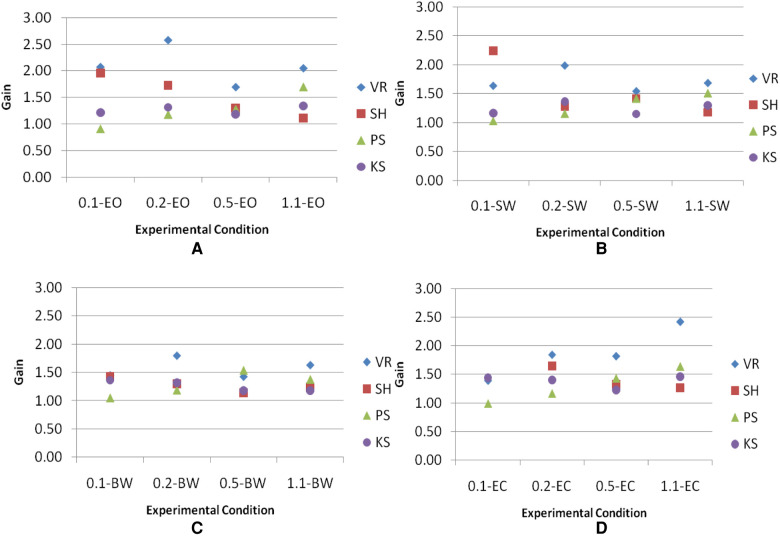
Gains of the head across frequencies in each visual condition. Visual conditions included here are (**A**) EO, (**B**) SW, (**C**) BW, and (**D**) EC.

Four visual inputs were applied to subjects, two of which were discordant with inertial motion (SW and BW), one was concordant (EO), and one had no conflict because there was no visual input (EC). Plots of gains across frequencies in each visual condition (Figure 9) showed that subjects VR and SH have more scattered gains and subjects PS and KS have gains close to 1.

The nonparametric statistical test was first used in analyzing the effect of frequencies and visual conditions on gains. The nonparametric technique can avoid assuming normal distribution or homogeneity of variance in the subjects involved. With four samples drawn from the same population, the Friedman two-way analysis of variance (ANOVA) by ranks was used first to test the significance of the difference of gains among all experimental conditions. *Null Hypothesis H*_0_ is that the different conditions in the experiment have no differential effect, whereas *Alternative Hypothesis H*_1_ is that the different conditions in the experiment have a differential effect. The established *significance level* is *α* = 0.05. The *sampling distribution* $\chi_{r}^{2}$ is distributed approximately as chi-square (25):

(9) $$\chi_{r}^{2}=\frac{12}{N\cdot k\cdot (k+1)}\sum_{\;j=1}^{k}(R_{i}{)}^{2}-3N\cdot (k+1)$$

*N* = number of subjects
*k* = number of conditions
*R_i_* = sum of ranks with the *j*th condition
$\sum_{\;j=1}^{k}$ directs one to sum the square of the sums of ranks over all *k* conditions

With *N* = 4 and *k* = 16, $\chi_{r}^{2}=12.19$. Reference to Table C (25) indicates that the result of $\chi_{r}^{2}$ is significant at between 0.7 and 0.5 levels of *p*-values. *p *≥ 0.5 is larger than *α* = 0.05. Therefore, the decision at this level is to accept *H*_0_. To increase the analytical power, gains were averaged among visual conditions at each frequency. The same Friedman test was used to evaluate the significance across frequencies. In this case, *Null Hypothesis H*_0_ is that the different frequencies have no differential effect, whereas *Alternative Hypothesis H*_1_ is that the different frequencies have a differential effect. The level of significance is chosen at *α* = 0.05. With *N* = 4 and *k* = 4, $\chi_{r}^{2}=3.0$. Reference to Table N (25) indicates that the result of $\chi_{r}^{2}$ is significant at *p* = 0.432. The *p*-value is larger than *α* = 0.05. Therefore, the decision is to accept *H*_0_. Overall, no significant difference in gains is believed to exist among visual conditions across frequencies. However, plots of gains across frequencies in each visual condition (Figure 9) showed that subjects VR and SH have more scattered gains and subjects PS and KS have gains close to 1. This may suggest that heads of subjects VR and SH are less restricted than those of subjects PS and KS.

**Figure 10:**
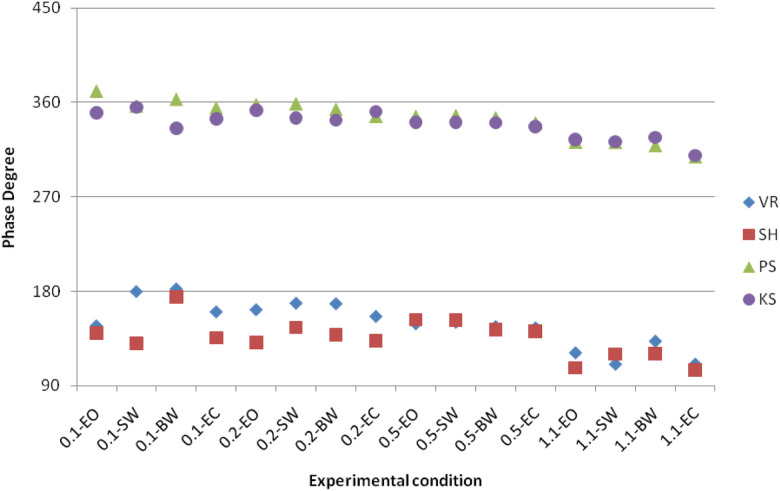
Phase of head acceleration relative to the sled.

Phase analysis converted the phase of head acceleration relative to the sled within the range from 0° to 360° in all experimental conditions. In the results shown in Figure 10, phases from two subjects VR and SH responded between 90° and 180° and two subjects PS and KS responded the phase close to 0°.

To gain more statistical power, a parametric statistical test was also used. A 4 × 4 two-way ANOVA with replication analyzed the effect of frequencies of motion profiles and visual conditions on gains. According to ANOVA results (Table 2), the *p*-values of frequency and visual conditions were 0.709 and 0.3556, respectively. Both were larger than the significance level of 0.05. Moreover, the *p*-value of interaction between these two factors was 0.987, much larger than the significance level. Therefore, there was no statistically significant difference in gains of head pitch acceleration among visual conditions and across the frequency. There was also no interaction between visual conditions and frequencies.

**Table 2:** Significance of frequencies and visual conditions and the interaction between them.

ANOVA						
Source of variation	*SS*	*Df*	MS	*F*	*p-*value	*F* crit
Frequency	0.330606	3	0.110202	0.463285	0.709236	2.798061
Visual	0.789752	3	0.263251	1.106695	0.355618	2.798061
Interaction	0.504749	9	0.056083	0.235771	0.987399	2.08173
Within	11.41782	48	0.237871			
Total	13.04292	63				

ANOVA, analysis of variance.

## Discussion

Head movement has been used as a measure of balance during active (voluntary) movement (i.e., walking). It has been previously reported that during passive (or involuntary) movement such as sudden base translation, head movement is significantly increased in the elderly, especially in those most susceptible to falls (26). During the active movement, the participants may need to initiate or be induced to slip or fall for the researchers to measure the range of head movement, which puts great demands on safety measures to prevent injury to the participants. On the other hand, the head movement can be examined during passive movement such as base translation, which is a relatively easier option with fewer demands on safety measures. The participants can be in a seated position, and different types of movement can be programmed. Following this rationale, we developed the sled system to investigate whether the head movement pattern and parameters can be used as a measure of balance in young adults.

Our experimental system was evaluated through a validation study. The subjects were tolerant of motion stimuli. There was no side effect after they were exposed to the level of stimuli. However, we are aware of some technical constraints. First, due to the limited length of the linear track, the system was unable to equalize the peak acceleration of the sled across all frequency points. Second, the linear track was essentially driven by an analog signal converted from a digital signal programmed by LabVIEW. To achieve the movement at certain amplitude, the signal amplitude has to be preset at the desired level for the programmable motor to execute the expected amplitude. Any mistake in the presetting may cause a rapid movement of the sled. In the future, we would like to improve our safety measures so that the motor drive system should be automatically shut down once the acceleration reaches its maximum level. We would also like to include other motion stimuli, such as ramp motion or sum-of-sines (SSN), that consists of relatively prime harmonics of a common base frequency (10) (repeated) in the future study.

Third, we did not specifically measure the head circumferences of participants. However, we made sure the HMD was adjusted until a comfortable fit was achieved for each subject. We did not find the literature explicitly stating that the head size can affect our study outputs. However, a recent study has reported that wearing the HMD affected Timed UP and GO (TUG) performance in younger and older adults as it increased the time taken to complete all TUG components (27). The subjects in our case study were in a seated position through all trials and did not mention that the lightweight HMD caused any discomfort, although the possibility that the added inertia of HMD may have confounded our results cannot be completely ruled out.

We also followed our IRB protocol to check the comfort level of participants and provided them with sufficient resting time between trials. No participant mentioned any discomfort. The participants did practice trials before the actual trials to get familiar with the experimental setup. Nevertheless, the pressure of HMD wear can increase flexion of the neck and trunk as subjects try to reach a slightly different head postural equilibrium and choose the best viewing angle to navigate through the environment.

Fourth, ideally, experimental runs should have been fully randomized. This means that the operating frequency of 0.1 Hz in this run may either not appear in the previous run or the next run. However, changing the frequency from 0.5 or 1.1 to 0.2 or 0.1 Hz needs to adjust not only the resolution of the motor from 1 to 7 but also the voltage of sinusoidal signals. It took 20 s or more to update the new resolution of the motor. To minimize order effects and save experimenting time, frequencies of motion stimuli were randomized between and within two frequency groups. The first group includes 0.1 and 0.2 Hz frequencies. The second group includes 0.5 and 1.1 Hz frequencies. Then, four visual inputs were randomly and equally selected for each experimental condition. Our arrangement of experimental conditions did not produce noticeable order effects based on the statistical analysis described in the Results section.

Self-motion can be induced by using dynamic visual input without concordant inertial motion, even if the visual-inertial conflict is present such as during sinusoidal translation (18). We created the conflict at frequencies equal to or less than 1.1 Hz. Although reports of perceived self-motion were not collected on all subjects, at least one subject remarked that he perceived diagonal side-to-side self-motion during the sideways visual condition.

As the major interest of study, we explored the head movement strategies used by these subjects during A-P sinusoidal translation. Gain plots may suggest that the heads of subjects VR and SH are less restricted than those of subjects PS and KS. Phase results indicate two head strategies were employed by seated subjects. The heads of two subjects were almost locked to sled motion, and the heads of two other subjects were more loosely stabilized and moved counter to the motion of the sled. These two strategies have been reported by Vibert et al. (28), wherein head-locked (i.e., “stiff”) subjects showed little translation of the head relative to the sled for the whole duration of motion stimuli when the trunk was fixed, whereas loose (i.e., “floppy”) subjects showed a large pitch of the head relative to the sled in the direction opposite to the sled movement. In addition, all subjects showed the drop-off phase at the highest frequency.

A sinusoidal acceleration of head suggests that the head–neck complex is indeed a second-order system with linear constant coefficient, which has been found in previous studies of head–neck control in humans (16) and anesthetized cats (29). Both studies have indicated that the kinematic response of the head depends on the inertia of the head and the viscosity and stiffness of the neck. The averaged head pitch acceleration increased as the maximum acceleration of the sled increased from 0.06*g* in the 0.1 Hz condition to about 0.12*g* in the 0.2 Hz condition. Greater neck stiffness and viscosity, as well as head inertia, should reduce the peak kinematic response of the head. The “stiff” strategy used by two subjects KS and PS might have increased the stiffness and viscosity of the neck. As a result, KS and PS had smaller gains and their head movement was in phase with the sled movement at the frequencies (<0.5 Hz) compared to SH and VR.

The head response of each participant was consistent over three test trials and four test conditions, although the amplitudes and phases of peak head movement displayed by the subjects varied and were distributed along a continuum between the two extremes corresponding to stiff and floppy participants. The variability of the subjects' head response could be because floppy participants were able to relax more than the stiff participants while anticipating the motion stimuli from the sled. We also ruled out the possibility that two different responses may rise from the variability in their level of anticipation of the stimulus. Subjects always had warnings in advance of the stimuli presence, and so all of them had similar anticipation of motion stimuli. Vibert et al. (28) suggested that the stiff participants may rely on visual cues, whereas the floppy participants may rely on the inertia of their head–neck ensemble to stabilize heads in space. However, two response strategies indicate that there may be more than one control mechanism influencing the stabilization of the head, which can include voluntary processes (22, 30).

The five-point harness in our study constrained the subject's trunk to the seat during passive locomotion. Our current findings show that the head pitch accelerations are greater than the sled acceleration almost in all subject conditions. We think that constraining the trunk may have impeded the ability of the trunk and neck to attenuate the momentum from the sled, ultimately increasing the amplitude and complexity of head acceleration (31).

In general, under no postural support during gait, acceleration amplitude was the greatest at the lower trunk and smallest for the head. The neck muscles help stabilize and reduce head acceleration. It was reported that an overall decrease in the ability of the lower trunk to attenuate the external oscillation leads to increases in the amplitude of vertical acceleration for the head when the trunk was singularly braced (31). Our study shows that these acceleration patterns also apply to the horizontal acceleration of the head during seated and passive locomotion.

The stability of the head–neck system is defined as a reduction of peak head velocity following the perturbation. An important component of head stabilization is the viscoelastic properties of the neck system. The stiffness in the neck needs to keep the head in static equilibrium against the force of gravity. In a situation where a perturbation of the head is applied, the brain could adjust muscle activation through co-contraction to alter neck stiffness and viscosity, and the large stiffness of the neck might reduce peak head angular velocity where a perturbation is applied (32). Neck stiffness and viscosity can be measured from the kinematic response of the head to an external perturbation. In the future, we would like to examine how neck joint stiffness and viscosity vary as a function of applied conditions and whether this can be beneficial for head stability by reducing peak head angular velocity.

## Data Availability

The original contributions presented in the study are included in the article/Supplementary Material, further inquiries can be directed to the corresponding author.
